# Acute Pancreatitis Secondary to Intragastric Balloon: A Case Report and Literature Review

**DOI:** 10.7759/cureus.45230

**Published:** 2023-09-14

**Authors:** Fakhreddin Al Refai, Sondos K Khalil, Sulafa K Khalil, Leena Saeed, Omar A Madani, Zahra B Yousif, Mustafa Ahmed

**Affiliations:** 1 Internal Medicine, Hamad Medical Corporation, Doha, QAT; 2 Medicine, University of Gezira, Khartoum, SDN; 3 Medicine, Hamad Medical Corporation, Doha, QAT; 4 Medicine, Qatar University, Doha, QAT

**Keywords:** pancreatitis, igb-induced pancreatitis, intragastric balloon, acute necrotizing pancreatitis, acute pancreatitis, igb complication

## Abstract

Intragastric balloon (IGB) is a common minimally invasive procedure used for obesity management and weight reduction. It can be used alone, sequentially, with concomitant therapies, or as a bridge to longer-term weight-loss interventions, such as bariatric surgery. Although the insertion procedure is easy and generally well tolerated by patients, a few complications can occur with varying degrees of severity ranging from mild to severe and life-threatening. Acute pancreatitis is a rare complication of IGB but has been reported in the literature. We present a case in which the patient had a history of IGB insertion complicated by acute pancreatitis. The diagnosis of acute pancreatitis due to the IGB insertion was made after excluding other possible causes of acute pancreatitis. The patient was hospitalized and managed conservatively.

## Introduction

Obesity is an alarmingly increasing global public health issue. The World Health Organization (WHO) expects around 176 million people to have their health compromised by 2025 due to weight-related problems [1]. It is associated with many complications and adverse health effects, including but not limited to morbidity from hypertension, dyslipidemia, type 2 diabetes, coronary heart disease, stroke, gallbladder disease, osteoarthritis, sleep apnea, and some malignancies. It is also associated with increased risk for all-cause and cardiovascular disease mortality [2].

Effective weight management is influenced by various intricate elements, including age, body mass index, patient lifestyle and habits, and sometimes patient preference of weight reduction methods. Several options for weight management are available, including a calorie-deficit diet, exercise, and behavioral modification, with or without pharmacological treatment. Bariatric surgery has been the mainstay of management in patients with morbid obesity; however, this does not apply to moderately obese patients, so other options of management are in use, including intragastric balloon (IGB) therapy [3].

We are reporting a case of acute pancreatitis secondary to IGB, a rare but important side effect of IGB placement.

## Case presentation

A 30-year-old lady presented to the emergency department complaining of epigastric and right upper quadrant pain after 41 days of IGB reinsertion.

Her past medical history was significant for gastroesophageal reflux disease, irritable bowel syndrome, and asthma controlled on inhalers. She was a smoker but denied alcohol intake. There was a family history of hypertension and diabetes in both of her parents. Her past surgical history included bilateral breast augmentation one year and six months back. She underwent endoscopic IGB insertion (Heliosphere Newtech IGB, Hélioscopie, Vienne, France; inflated with 600 cc of air) eight months ago for morbid obesity with a BMI of 35. After failure to lose weight with dietary modification, removal and reinsertion were done 41 days prior to presentation.

Upon questioning, she reported experiencing an abrupt onset of abdominal pain. The pain began in the epigastric area, was constant in nature, radiating to the back, associated with nausea and vomiting, with no aggravating or relieving factors. She had been constipated, with her last bowel movement two days ago. No yellowish discoloration of the sclera, no difficulty swallowing, anorexia, or weight loss was noted. Earlier on the same day, she had visited the emergency department with a complaint of cough, fever, and headache. She was diagnosed with respiratory tract infection, prescribed amoxicillin/clavulanate, and was sent home.

On examination, she was febrile (38.1°C), normotensive at 120/69 mmHg, with a pulse rate of 105 beats/minute, respiratory rate of 18 breaths/minute, and maintained saturation on room air. She was conscious, alert, and oriented to time, place, and person. Abdominal examination revealed mild tenderness in the epigastric area, no hepatomegaly or splenomegaly, and bowel sounds were normal. Precordium, chest, neurological, and musculoskeletal examinations were normal.

Initial laboratory testing showed normal complete blood count and renal and liver function tests. Amylase and lipase were significantly elevated, with mild elevation in C-reactive protein (Table 1).

**Table 1:** Initial laboratory tests

Test	Result	References
Amylase	218 U/L	8.0-51.0 U/L
Lipase	565 U/L	8-78 U/L
C-reactive protein	16 mg/dL	0-5 mg/dL

**Figure 1:**
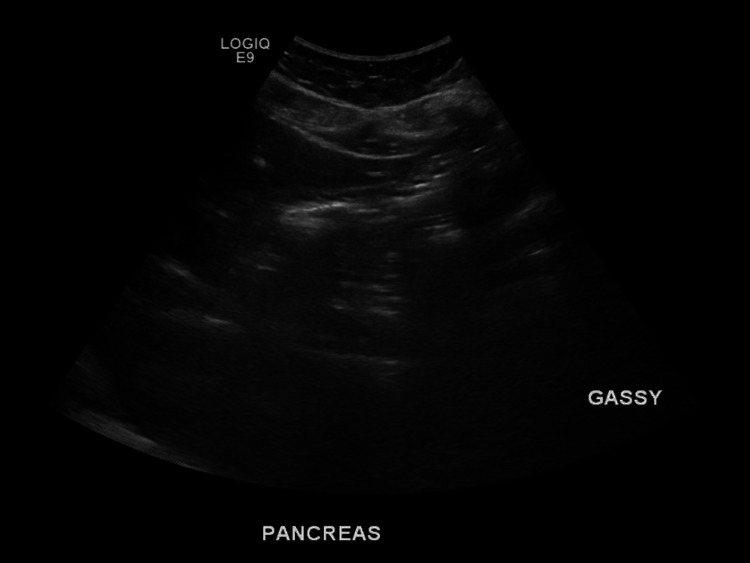
Abdominal ultrasound

Abdominal ultrasound showed a normal gallbladder without stones and a normal common bile duct diameter. The pancreas was obscured by bowel gas (Figure 1).

She was hospitalized and was managed with intravenous fluids, analgesia, and antiemetics. She significantly improved with pain subsidence and was discharged home two days later.

## Discussion

IGB is a non-surgical, temporary weight loss therapy [4]. Garren-Edwards gastric bubble (GEGB) was the first recognized IGB in the United States by the Food and Drug Administration (FDA); however, it had unsatisfying outcomes regarding weight loss with many consequential complications [5-7]. Due to its numerous adverse events and early balloon deflation, first-generation IGBs, including GEBG, were quickly deserted in the 1980s [8]. The newer fluid-filled balloons with lower rates of complications are currently the mainstay intervention for weight-loss management approved by the FDA [9].

IGB promotes weight loss through various mechanisms. It acts as a bezoar that preloads the stomach leading to early satiety and decreased food intake [10]. A neurohormonal mechanism has also been suggested when combined with cholecystokinin intake [11]. Mion et al. described how IBG placement in the stomach results in noticeable ghrelin inhibition and delayed gastric emptying [12].

IGB has been associated with different complications, including minimal abdominal pain, intractable vomiting, gastroesophageal reflux, gastric ulcers and perforation, obstruction, and the most frequent being balloon removal due to intolerance [13].

Although rare, acute pancreatitis as an adverse event of IGB placement can occur. Between 2008 and 2012, only three cases were reported; this number rose to 10 cases between 2012 and 2014 [14], after which several cases were reported. Fluid-filled balloons like Orbera and ReShape were approved by the FDA in 2015; however, the FDA issued a safety warning illustrating their association with acute pancreatitis in February 2017. Cases linked to air-filled cases have also been published but the association was considered unlikely [15].

We are reporting a 30-year-old female who presented with symptoms of acute pancreatitis following IGB insertion. The period for developing acute pancreatitis following IGB insertion varies but mostly is between one and eleven months, and most cases occur in balloons inflated with 400-700 ml [16]. Our patient falls within the category of most reported cases, as she developed symptoms 41 days after removal and reinsertion of the balloon.

Most cases had mild to moderate symptoms ranging from minimal abdominal pain to nausea and vomiting and moderate severity abdominal pain; however, severe cases were reported. Vongsuvanh et al. reported a case of severe acute necrotizing pancreatitis with gastric ischemia and hepatic portal venous gas [17]. On the other hand, Halpern et al. reported an unusual case of asymptomatic elevated pancreatic enzymes secondary to IGB; this is thought to be the first asymptomatic IGB-induced acute pancreatitis [18].

The mechanism of IGB-associated pancreatitis is not fully understood; however, different mechanisms in the literature were proposed based on CT and endoscopic findings. Compression of the pancreas by a distended stomach has been the mechanism in most cases [19]. Balloon catheter migration to the duodenum has also been reported [17]. Interestingly Ağca et al. reported a case due to whole balloon migration to the duodenum [14].

Diagnosis can be made the same as in acute pancreatitis of any etiology, the presence of two of the three following criteria confirms the diagnosis: epigastric abdominal pain, elevated serum lipase three times or greater than the upper limit of normal, and characteristic findings for acute pancreatitis on imaging. Our patient was diagnosed based on a clinical and biochemical basis. It is worth mentioning if clinical and biochemical evidence of acute pancreatitis is present; imaging need not be done, nonetheless, it may be indicated if whole balloon migration is suspected, which can guide the management decision.

Two options for the management have been implemented. Early removal of the balloon seemed to be the management of choice for most physicians, endoscopic removal was the preferred method [20,21], and surgical removal should be considered if the balloon is endoscopically inaccessible [19,17]. The second option applied later was conservative management, which had a good outcome [22]. We propose a trial of conservative management in uncomplicated cases with an in-place balloon before attempting removal to reduce unnecessary interventions.

## Conclusions

Acute pancreatitis in the setting of IGB is a rare but possible complication. Clinical manifestation may mimic common side effects of the procedure like nausea, vomiting, and abdominal pain making the diagnosis challenging. We caution physicians to have high suspicion when the clinical picture is suggestive and to investigate pancreatitis to aid in early diagnosis of the condition. Imaging is not generally indicated if both clinical and biochemical evidence of the disease are present; however, we recommend imaging studies prior to management initiation to determine balloon location, as it can guide management decisions. Finally, we suggest conservative management in clinically and radiologically appropriate cases before attempting balloon removal to minimize unnecessary interventions.
